# Divide-and-Attention Network for HE-Stained Pathological Image Classification

**DOI:** 10.3390/biology11070982

**Published:** 2022-06-29

**Authors:** Rui Yan, Zhidong Yang, Jintao Li, Chunhou Zheng, Fa Zhang

**Affiliations:** 1 High Performance Computer Research Center, Institute of Computing Technology, Chinese Academy of Sciences, Beijing 100045, China; yanrui20b@ict.ac.cn (R.Y.); yangzhidong19s@ict.ac.cn (Z.Y.); jtli@ict.ac.cn (J.L.); 2 University of Chinese Academy of Sciences, Beijing 101408, China; 3 School of Artificial Intelligence, Anhui University, Hefei 230093, China

**Keywords:** pathological image classification, attention mechanism, convolutional neural network, knowledge embedding

## Abstract

**Simple Summary:**

We propose a Divide-and-Attention network that can learn representative pathological image features with respect to different tissue structures and adaptively focus on the most important ones. In addition, we introduce deep canonical correlation analysis constraints in the feature fusion process of different branches, so as to maximize the correlation of different branches and ensure that the fused branches emphasize specific tissue structures. Extensive experiments on three different pathological image datasets show that the proposed method achieved competitive results.

**Abstract:**

Since pathological images have some distinct characteristics that are different from natural images, the direct application of a general convolutional neural network cannot achieve good classification performance, especially for fine-grained classification problems (such as pathological image grading). Inspired by the clinical experience that decomposing a pathological image into different components is beneficial for diagnosis, in this paper, we propose a **D**ivide-and-**A**ttention **Net**work (**DANet**) for Hematoxylin-and-Eosin (HE)-stained pathological image classification. The DANet utilizes a deep-learning method to decompose a pathological image into nuclei and non-nuclei parts. With such decomposed pathological images, the DANet first performs feature learning independently in each branch, and then focuses on the most important feature representation through the branch selection attention module. In this way, the DANet can learn representative features with respect to different tissue structures and adaptively focus on the most important ones, thereby improving classification performance. In addition, we introduce deep canonical correlation analysis (DCCA) constraints in the feature fusion process of different branches. The DCCA constraints play the role of branch fusion attention, so as to maximize the correlation of different branches and ensure that the fused branches emphasize specific tissue structures. The experimental results of three datasets demonstrate the superiority of the DANet, with an average classification accuracy of 92.5% on breast cancer classification, 95.33% on colorectal cancer grading, and 91.6% on breast cancer grading tasks.

## 1. Introduction

Accurate cancer classification and grading can help doctors make overall treatment plans and predict prognosis. Pathologists examine the Hematoxylin-and-Eosin (HE)-stained pathological sections to complete the pathological diagnosis, which is usually the gold standard. Hematoxylin is an alkaline dye that can stain basophilic tissue structures (such as the chromatin in the nucleus) as purple–blue; in contrast to hematoxylin, eosin is an acidic dye that can stain eosinophilic tissue structures (such as the components in the cytoplasm and extracellular matrix) as red. Therefore, the pathologist can visually distinguish the nuclei part from the non-nuclei part.

Although deep-learning methods have greatly improved the performance of pathological image classification [1], they seldom exploit the inherent characteristics of pathological images, such as the irregularity of tumor cells and tissues, and the inter- and intra-nuclear color variations. Therefore, the direct application of general deep-learning methods cannot achieve satisfactory classification results; especially the fine-grained classification problems (such as pathological image grading) cannot be handled well.

The algorithm performance can be further improved with the help of medical knowledge [2], but how to embed medical knowledge into the end-to-end learning of the algorithm still faces huge challenges. Some current work has been explored along this line, and one of the best practices is to leverage the medical anatomy knowledge [3,4,5]. However, the medical anatomy knowledge cannot be well reflected in pathological images. This is because pathological images reflect microscale phenotypes (tissue and cell levels), which are too microscopic with too few specific rules to follow. Especially cancer occurrence and development make it even more irregular.

In this paper, to leverage the medical staining knowledge belonging to HE-stained pathological image, we propose a Divide-and-Attention Network (DANet) for pathological image classification. The DANet utilizes a deep-learning method to decompose a pathological image into nuclei and non-nuclei parts. With such decomposed pathological images, the DANet first performs feature learning independently in each branch, and then focuses on the most important feature representation through the branch selection attention. In this way, the DANet can learn more representative features with respect to different tissue structures and adaptively focus on the most important ones, thereby improving classification performance. This is similar to the traditional divide-and-conquer algorithm design idea. A complex problem is first decomposed into sub-problems with the help of prior knowledge, and then the answers to the sub-questions are fused to yield the final result. For the DANet method, the sub-question answers correspond to the feature representation independently learned by each branch of the DANet, and the fusion of sub-question answers corresponds to the attention mechanism of the DANet.

In addition, we introduce deep canonical correlation analysis (DCCA) in the feature fusion process of different branches. The basic idea behind DCCA is to maximize the correlation of different multidimensional variables and extract common components. In the DANet, the DCCA constraints can play the role of branch fusion attention in the feature fusion of different branches. For example, after the fusion of the main branch (original image) and the down branch (containing only the nuclei) under DCCA constraints (nuclei attention), the obtained middle branch can pay more attention to the “nuclei-related features”.

We conducted experiments on breast cancer classification, colorectal cancer grading, and breast cancer grading, and achieved an average classification accuracy of 92.5%, 95.33%, and 91.6%, respectively. The experimental results show that the DANet achieved competitive performance and could play a fundamental role in HE-stained pathological image analysis.

## 2. Related Works

### 2.1. Pathological Image Classification

Pathological image classification methods can be summarized into three categories from the following perspectives: Patch-wise (hundreds × hundreds of pixels) methods, image-wise (thousands × thousands of pixels) methods, and WSI-wise (tens of thousands × tens of thousands of pixels) methods. Patch-wise pathological image classification methods are the basis of the image-wise and WSI-wise methods. In this work, we only focus on the patch-wise methods. Spanhol et al. [6] published BreaKHis, a pathological image classification dataset of benign and malignant breast cancer. On the basis of this dataset, Spanhol et al. [6] used the AlexNet network and a variety of fusion strategies to perform patch classification, and the classification accuracy was increased by 6% compared with traditional machine-learning algorithms. Also based on the BreaKHis dataset, Bayramoglu et al. [7] used a deep-learning method that did not depend on image magnification, and the accuracy of patch classification was about 83%. Araujo et al. [8] further studied the four-classification of breast cancer pathological images (normal tissue, benign tissue, in situ carcinoma, and invasive carcinoma). They first used a convolutional neural network (CNN) to extract the features of the patch, and then used the support vector machines (SVM) algorithm to classify. The average patch-wise classification accuracy was 77.8%. Jiang et al. [9] proposed a novel CNN with a SE-ResNet module, which is an improvement on the combination of the residual module and the squeeze-and-excitation block. Their method achieved an accuracy between 90.66% and 93.81% for the four-class pathological image classification.

Pathological image grading is a fine-grained classification task. The difference between pathological images of different grades is very subtle, and general deep-learning methods cannot handle such tasks well. Therefore, for different pathological image grading tasks, researchers have designed different network structures and methodological frameworks [10,11,12]. Wan et al. [10] used a cascade method to distinguish low-, intermediate-, and high-level breast cancers. They first separately obtained semantic-level features extracted by a CNN, pixel-level texture features, and object-level structural features, and then combined all these image features for breast cancer grading. Yan et al. [11] proposed a breast cancer grading network (NANet) that can focus on nuclei-related features through end-to-end learning. The current colorectal cancer grading method ignores the importance of the tissue microenvironment, which can be evaluated by cell-level information and gland morphology. To overcome these shortcomings, Zhou et al. [13] proposed a Cell Graph Convolutional Neural Network (CGC-Net). The CGC-Net converts a pathological image into a graph, in which each node is represented by a cell nucleus in the original pathological image, and the edges between nodes (cell interaction) are represented by node similarity. The results show that compared with the traditional patch-based method, modeling the image as a graph can effectively process pathological images with larger pixels, and can model complex tissue microenvironments. In addition, there is also a semi-manual grading method, which first decomposes the pathological image grading task into some easily identifiable indicators according to medical knowledge, and then separately calculates these indicators to obtain the final result. For example, when grading breast cancer, we can first obtain the three indicators of Nottingham Grading System (NGS): (1) nucleus pleomorphism [14], (2) tubular formation [15], and (3) mitotic count [16], and then integrate these three indicators to decide the final grading diagnosis. A preliminary pathological image classification method that we proposed (Decomposition-and-Fusion Network, DFNet) was published as a conference paper [17]. On the basis of the DFNet, we put forward new contributions in branch selection attention and branch fusion attention to learn more representative pathological features.

### 2.2. DCCA in Multi-Modal Fusion

Canonical correlation analysis (CCA) [18] is a widely used method in statistics to measure the linear relationship between two multidimensional variables. Let ($X_{1},X_{2}$) denote random vectors. The CCA finds pairs of linear projections of the two variables that are maximally correlated:

(1) $$\left(w_{1}^{*},w_{2}^{*}\right)=\underset{W_{1},W_{2}}{\arg\max}\;corr\left(w_{1}^{⊺}X_{1},w_{2}^{⊺}X_{2}\right)\cdot x$$

Hardoon et al. [19] introduced CCA to machine learning, and Andrew et al. [20] further proposed a deep neural network extension of CCA, termed DCCA, which computes representations of the two modes by passing them through multiple fully connected layers. Adding DCCA constraints to multi-modal fusion can maximize the correlation of different modes and extract common components [21,22,23,24]. For example, Liu et al. [21] introduced DCCA to a multi-modal emotion recognition task and Sun et al. [22] proposed learning multi-modal (text, audio, and video) embeddings using DCCA for the improvement of sentiment detection.

### 2.3. Attention Mechanism

Inspired by human attention, the attention mechanism has been developed in deep-learning methods [25]. Human vision quickly scans the global image to obtain the region of interest, and then focuses on this area, thereby suppressing other irrelevant information. There are many excellent deep-learning methods based on attention mechanisms, such as the SENet [26], Weight Excitation [27], CBAM [28] and Dual Attention Network [29]. The self-attention mechanism is a variant of the attention mechanism, which is good at capturing the internal correlation between input data. The Vision Transformer (ViT) [30] is an excellent method for applying self-attention mechanisms to the computer vision field. The research performed model pre-training based on a large amount of data that are then transferred to multiple image classification benchmark datasets. The results show that the ViT model can obtain results comparable to the current state-of-the-art CNN methods, while the computing resources required for its training are greatly reduced. Based on the ViT, the breakthrough progress of the Swin Transformer [31], Pyramid Vision Transformer [32], and Neighborhood Attention Transformer [33] on multiple tasks makes it possible to use a ViT-like model as an alternative to a CNN. In the field of multi-instance learning, the attention-based pooling operation [34] significantly improves the performance of the algorithm. Inspired by this work, we adopted an attention mechanism in the multi-branch fusion stage.

### 2.4. Nuclei Segmentation

Nuclei segmentation is used in the pathological image decomposition part of the proposed method, so we also briefly introduce the related deep-learning-based nuclei segmentation methods. The robustness of the traditional nuclei segmentation methods [35] is poor due to the inter- and intra-nuclear color variations in crowded and chromatin-sparse nuclei. Methods based on deep learning, especially methods based on CNN, can obtain excellent results from the challenging images of nuclei segmentation because they can adaptively learn from big data end-to-end. Chen et al. [36] presented a deep contour-aware network that integrates multi-level contextual features to segment glands and nuclei. They define the segmentation problem as a multi-instance classification problem by explicitly harnessing the complementary appearance information and contour information. Kumar et al. [37] introduced a large HE-stained pathological image dataset with more than 21,000 annotated nuclear boundaries; they also proposed a CNN-based segmentation method that lays special emphasis on identifying the nuclear boundaries. With the help of the information encoded within the vertical and horizontal distances of nuclear pixels to their centers of mass, Graham et al. [38] presented a Hover-Net for simultaneous nuclei segmentation and classification. A more comprehensive review of nuclei segmentation methods can be found in the review literature [35].

## 3. Methods

In this section, we first describe the pathological image decomposition part (that is, the nuclei segmentation network) in Section 3.1, then describe the pathological image classification part (that is, the architecture of the DANet) in Section 3.2. In Section 3.3, we introduce one of the key modules in DANet: the branch selection attention module. Finally, we introduce the proposed loss function, especially the introduced deep canonical correlation analysis loss, in Section 3.4.

### 3.1. Pathological Image Decomposition Part

The main purpose of HE staining is to distinguish the nuclei and non-nuclei parts (cytoplasm, extracellular matrix). With simple color decomposition, it is impossible to achieve medically meaningful pathological image decomposition due to the large inter- and intra-nuclear color variations. If we can achieve the nuclei segmentation task, then the goal of decomposing HE-stained images can also be achieved.

We used DeepLabV3+ [39] to perform nuclei segmentation. DeepLabV3+ proposes an encoder–decoder network structure, in which the encoder is able to encode multi-scale contextual information by using the Atrous Spatial Pyramid Pooling (ASPP) module, and the decoder can capture sharper object boundaries by gradually recovering the spatial information. This advantage of DeepLabV3+ is particularly helpful in solving the hurdles of the nuclei segmentation task, such as the nuclei size difference and nuclei boundary overlap.

**Figure 1:**
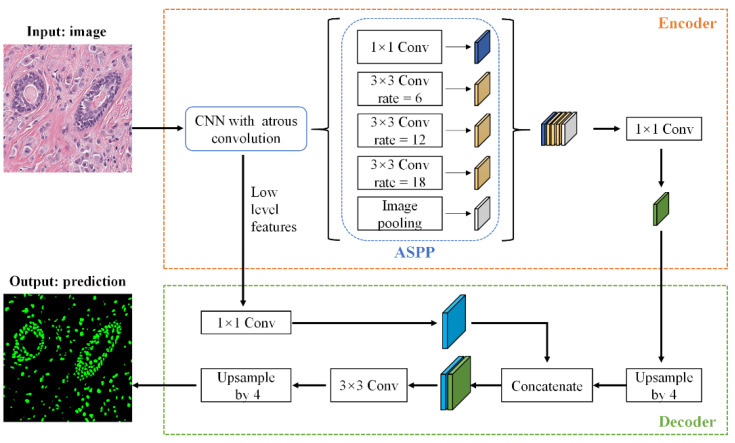
The architecture of DeepLabV3+ applied to nuclei segmentation.

Given a pathological image $I_{main}$, the output of DeepLabV3+ is the nuclei segmentation mask *M*. The network structure of DeepLabV3+ is shown in Figure 1. In the encoder stage, DeepLabV3+ first uses Xception as the network backbone to perform feature extraction on $I_{main}$, and then feeds it into the ASPP module. The ASPP effectively captures multi-scale features through five parallel branches: one 1 × 1 convolution, three 3 × 3 convolutions with atrous rates = (6, 12, 18), and a global average pooling. The resulting feature maps from all the five branches are then concatenated and passed through another 1 × 1 convolution to generate the encoder output. In the decoder stage, the encoder output is firstly upsampled by 4 times, and then concatenated with the low-level features extracted from the network backbone Xception. The purpose of the 1 × 1 convolution on low-level features is to reduce the number of channels. After the concatenation, we apply a 3 × 3 convolution to refine the features followed by another upsampling by a factor of 4. Finally, the decoder outputs the prediction result.

### 3.2. Pathological Image Classification Part

The proposed DANet has three inputs ($I_{main},I_{down},I_{top}$). The input to the main branch is the original pathological image $I_{main}$, and the inputs to the two auxiliary branches are two images: one image $I_{down}$ containing only the nuclei, and the other image $I_{top}$ containing the non-nuclei, respectively. The relationships between the three inputs are:

(2) $$I_{down}=M*I_{main},\;$$

(3) $$I_{top}=I_{main}\;-\;I_{down}.\;$$

**Figure 2:**
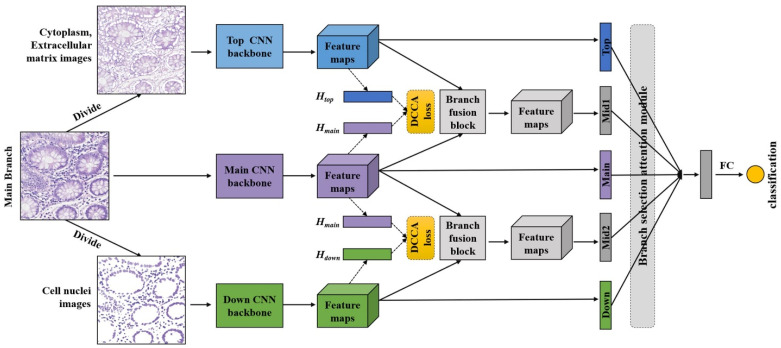
The overall network structure of the DANet. The proposed DANet has three inputs: the original pathological image (main branch), the nuclei image (down branch), and the non-nuclei image (top branch). Two middle branches are obtained through the branch fusion block and branch fusion attention (DCCA loss). After the independent feature extraction of a single branch and feature fusion of different branches, we use GAP to compress the feature maps obtained from the five branches into five feature vectors. Finally, the five feature vectors are passed through the branch selection attention module and the fully connected network to obtain the final classification result. The loss function for the DANet is defined as the combination of the cross-entropy loss and DCCA loss (indicated in yellow).

The three branches (top, main, down) of the DANet first perform feature extraction independently. Then, the output feature maps of these three branches are fed to the branch fusion blocks, deriving two middle branches. At the end of the five branches, we apply global average pooling (GAP) to obtain five one-dimensional feature vectors. These five feature vectors are fed into the branch selection attention module to adaptively select the most important feature representation derived from the five branches. In this way, the DANet can learn more representative features with respect to different tissue structures and adaptively focus on the most important ones. The resulting features from the branch selection module are then passed through the fully connected neural network to yield the final classification result. The overall network structure of the DANet is shown in Figure 2.

We use Xception [40] as the CNN backbone for independent feature representation learning. Due to its lightweight design, the total parameter of Xception is about 23 M, which gives rise to the total parameter of the DANet model of about 95 M. Although the number of parameters of the DANet has increased, there is still a 32% reduction compared to VGG16 (139 M).

In the branch fusion block, the output feature maps ($X_{top}$ and $X_{down}$) extracted from each auxiliary branch are concatenated with the output feature map ($X_{main}$) extracted from the main branch, respectively. Next, the 1 × 1 and 3 × 3 convolution operations follow. The purpose of the 1 × 1 convolution operation is to fuse information between channels and also to compress feature maps [41]. The purpose of the 3 × 3 convolution operation is to perform feature learning again after fusion. The formulas are denoted as follows:

(4) $$X_{k}=f_{Xcep}\left(I_{k}\right),\;k\;\in \;\left\{top,main,down\right\},\;$$

(5) $$X_{mid1}=f_{mid1}\left(X_{main},X_{top}\right),\;$$

(6) $$X_{mid2}=f_{mid2}\left(X_{main},X_{down}\right),\;$$

where *X* represents the feature maps and *I* represents the input images. $f_{Xcep}\left(.\right)$ represents the extraction of last feature maps from Xception backbone. $f_{mid}\left(.\right)\;$ represents the fusion block. In this work, the dimension of $X_{k}$ is (10, 10, 512). After the fusion block, we use GAP to compress the feature maps obtained from the five branches into five feature vectors:

(7) $$V_{k}=f_{GAP}\left(X_{k}\right),\;k\;\in \;\left\{top,mid1,main,mid2,\;down\right\}.\;$$

Finally, the five feature vectors $V_{k}$ are passed through the branch selection attention module $f_{att}\left(.\right)$ and the fully connected network $f_{FCN}\left(.\right)$ to obtain the final classification result.

(8) $$Y=f_{FCN}\left(f_{att}\left(V_{top},\;V_{mid1},\;V_{main},\;V_{mid2},\;V_{down}\right)\right),\;$$

where *Y* represents the image label. The attention module and loss function are described in detail below.

### 3.3. Branch Selection Attention Module

The DANet first performs feature learning independently in each branch. Specifically, in addition to the general feature representation learned by the main branch, the DANet also learns more feature representations: the top branch learns “non-nuclei-related features”, the down branch learns “nuclei-related features”, and the two middle branches further learn to emphasize the features related to the nuclei or non-nuclei on the basis of the general features. Since different classification tasks have different requirements for the learned feature representation, we hope that the network can adaptively focus on the required feature representation through learning. Inspired by the attention-based pooling operation in multi-instance learning [34], we propose the branch selection attention module in the DANet. In this way, the DANet is able to learn features with respect to different structures and adaptively focus on the most important ones, thereby improving classification performance.

**Figure 3:**
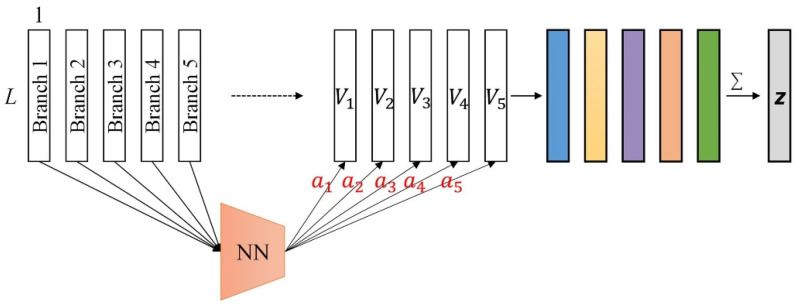
The branch selection attention module in the DANet. The fully connected neural network (NN) represents: FC(128)→tanh→FC(64)→softmax.

The five branches of the DANet output five one-dimensional feature vectors: $V_{k},\;\mathrm{where}\;V_{k}\;\in \;{\mathbb{R}}^{L}\;\mathrm{and}\;k\;\in \;\left\{top,mid1,main,mid2,\;down\right\}$. The output of the branch selection attention module can be calculated as follows:

(9) $$z=\sum^{\;}\left(a_{k}V_{k}\right),$$

(10) $$a_{k}=\frac{\exp\left\{w^{⊺}\tanh\left(QV_{k}^{⊺}\right)\right\}}{\sum_{j}^{k}\exp\left\{w^{⊺}\tanh\left(QV_{j}^{⊺}\right)\right\}},$$

where $a_{k}$ represents the attention weight, and $w\;\in {\mathbb{R}}^{5\times 1}$ and $Q\;\in {\mathbb{R}}^{5\times L}$ are learnable parameters. In this paper, we take L=512. See Figure 3 for the detailed network structure of the branch selection attention module.

### 3.4. Loss Function

The loss function for the DANet is defined as the combination of the cross entropy (CE) loss and DCCA loss:

(11) $$L_{CE}=-\frac{1}{m}\sum_{i=1}^{m}\sum_{k=1}^{k}q_{k}^{m}\log\left(p_{k}^{m}\right),\;$$

(12) $$L_{DANet}=\lambda_{1}L_{CE}+\lambda_{2}L_{DCCA},\;$$

where $q_{k}^{m}$ and $p_{k}^{m}$ indicate the ground truth and prediction probability of the *m*th image for *k*th class, and $\lambda_{1}\;$ and $\lambda_{2}$ are hyperparameters. Here, we introduce deep canonical correlation analysis (DCCA) [20] for the feature fusion of different branches. The basic idea behind DCCA is to maximize the correlation of different multidimensional variables and extract common components. In the DANet, the DCCA constraints act as the branch fusion attention in the feature fusion of different branches. For example, the middle branch obtained after the fusion of the main branch (containing the original image) and the down branch (containing only the nuclei) under DCCA constraints can pay more attention to the “nuclei-related features”, which are important in certain HE-stained pathological image classification tasks (such as breast cancer grading).

We take the feature fusion of the main and down branches as an example to illustrate DCCA, and the feature fusion of the main and top branches is exactly the same. Let $I_{1}$ be the input for the main branch and $I_{2}$ be the input for the down branch. After the following nonlinear transformation, we can obtain the output of the two branches: $H_{1}\;\mathrm{and}\;H_{2}$, which are formulated as:

(13) $$H_{k}=f_{FC}\left(f_{GAP}\left(f_{Xcep}\left(I_{k}\right)\right)\right),\;k\;\in \;\left\{1,2\right\}.\;$$

Let $W_{k}$ denote all the parameters for the non-linear transformations. The goal of DCCA is to jointly learn the parameters $W_{k}$ to maximize the correlation of $H_{1}$ and $H_{2}$:

(14) $$W_{k}^{*}=\underset{W_{k}}{\arg\;\max}\;corr\left(H_{1},H_{2}\right).\;$$

To find $W_{k}^{*}$, we follow the way used by Andrew et al. [20] to express the solution to this objective. Let $H_{k}\;\in \;{\mathbb{R}}^{N\times d}$ be matrices whose columns are the feature representation vector. Here, *N* is the batch size, and *d* is the dimension of the extracted feature representation vector. Let $\bar{H_{k}}$ = $H_{k}^{⊺}-\frac{1}{N}H_{k}^{⊺}1$ be the centered output matrix, and define ${\sum^{\;}}_{12}=\frac{1}{N-1}\bar{H_{1}}\;\bar{H_{2}^{⊺}}$ and ${\sum^{\;}}_{11}=\frac{1}{N-1}\bar{H_{1}}\;\bar{H_{1}^{⊺}}+r_{1}I$. Here, $r_{1}$ is a regularization parameter (similar to ${\sum^{\;}}_{22}$). In this paper, we take *m* = *d*, then the total correlation can be denoted as:

(15) $$T={\sum^{\;}}_{11}^{-1/2}{\sum^{\;}}_{12}{\sum^{\;}}_{22}^{-1/2},\;$$

(16) $$corr\left(H_{main},H_{down}\right)={\left(tr\left(T^{⊺}T\right)\right)}^{\frac{1}{2}},\;$$

therefore, the DCCA loss is to minimize $L_{DCCA}$:

(17) $$L_{DCCA}=-corr\left(H_{main},H_{down}\right)-corr\left(H_{main},H_{top}\right).\;$$

## 4. Results and Discussion

### 4.1. Evaluation Metrics and Training Details

The existing works mainly use accuracy and AUC metrics to evaluate the performance of HE-stained pathological image classification methods, and we also followed this tradition. The accuracy, sensitivity, specificity, and F-score (the harmonic mean of precision and sensitivity) metrics can be defined as follows:

(18) $$\mathrm{Accuracy}\;=\frac{TP+TN}{TP+TN+FP+FN},\;$$

(19) $$\mathrm{Sensitivity}\;=\frac{TP}{TP+FN},\;$$

(20) $$\mathrm{Specificity}\;=\frac{TN}{TN+FP},\;$$

(21) $$F-\mathrm{score}=\frac{2TP}{2TP+FP+FN},\;$$

where *TP*, *TN*, *FP*, and *FN* represent the True Positive, True Negative, False Positive, and False Negative, respectively.

We randomly selected 80% of the dataset to train and validate the model, and the remaining 20% was used for testing. The model parameters in $L_{DANet}$ are given by $\lambda_{1}$ = $\lambda_{2}$ = 0.5. We adopted the Adam algorithm with β1 = 0.9 and the learning rate *lr* = 0.001 to train the DANet. The regularization constant $r_{1}$ = $r_{2}$ = 0.001. For DeepLabV3+, when the training steps were 100,000, the best experimental results were achieved. The values of the atrous rates we used were 6, 12, and 18, and the output stride we adopted was 16.

### 4.2. Datasets and Preprocessing

#### 4.2.1. Breast Cancer Classification Dataset

**Figure 4:**
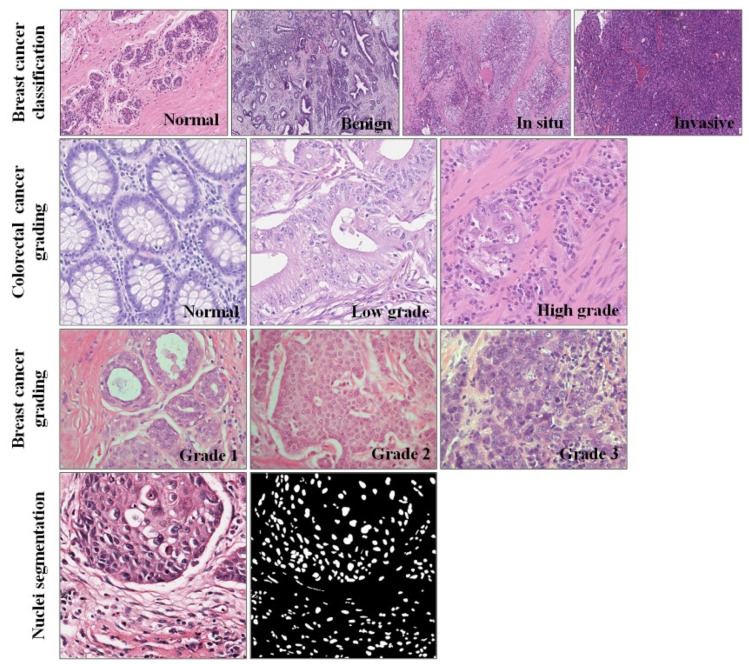
Examples of the four datasets used in the experiments.

The publicly available dataset of breast cancer (BC) pathological image classification proposed by Yan et al. [42] contains 3771 high-resolution (2048 × 1536 pixels) images. Among them, the number of pathological images of normal, benign, in situ carcinoma and invasive carcinoma is 299, 1106, 1066, and 1300, respectively. All the pathological images are divided into non-overlapping 512 × 512 patches. Due to the data imbalance, we separately enhanced the normal category. After preprocessing, the final BC dataset included 45,660 patches, which consisted of 10,000 normal, 10,672 benign, 11,630 in situ, and 13,358 invasive patches. An example of the dataset is shown in Figure 4. Another widely used dataset was released by the grand challenge on Breast Cancer Histology images (BACH) [43]. The dataset contains four categories, each with 100 pathological images. Most of the published papers are based on this dataset. Therefore, we also made comparisons with the proposed state-of-the-art methods based on this dataset.

#### 4.2.2. Colorectal Cancer Grading Dataset

For the task of colorectal cancer grading, Awan et al. [44] proposed a dataset consisting of 139 high resolution (4548 × 7548 pixels) pathological images, comprising 71 normal, 33 low-grade, and 35 high-grade images. Similar to the preprocessing steps of the breast cancer classification dataset above, all the images of 4548 × 7548 pixels were divided into patches of 512 × 512 pixels, and the patch-level label was derived from the image-level label. To make the divided image size exactly an integer multiple of 512, we first resized the original images to 4608 × 7680. Thereby, an original pathological image could be divided into 135 non-overlapping patches (512 × 512 pixels). After cutting, the colorectal cancer dataset included 15,303 patches, which consisted of: 6716 normal, 4348 low-grade and 4239 high-grade cancer patches.

#### 4.2.3. Breast Cancer Grading Dataset

Currently, there are mainly two public datasets on breast cancer pathological image grading, of which the largest amount of data is proposed by Yan et al. [11]. A total of 3644 high-resolution (1000 × 1000 pixels) breast IDC images are included. Among them, the number of pathological images of Grade1, Grade2 and Grade3 are: 961, 1121, 1562. Unlike the other two datasets we used, the image resolution of this dataset was not too high, so we did not perform non-overlapping cutting. Instead, regular data enhancements, such as zoom, flip, constant, brightness, contrast, crop, etc., were carried out. After data augmentation, the BC grading dataset included 32,820 pathological images, which consisted of: 9610 Grade1, 11,210 Grade2 and 12,000 Grade3 images. The experiment was conducted on this dataset. Another widely used dataset was proposed by Kosmas et al. [45]. The dataset includes 300 pathological images, which consist of: 107 Grade1 images, 102 Grade2 images, and 91 Grade3 images, all with a resolution of 1280 × 960. Most of the published papers are based on this dataset. Therefore, we also made comparisons with the proposed state-of-the-art methods based on this dataset.

#### 4.2.4. Nuclei Segmentation Dataset

The nuclei segmentation dataset released by Kumar et al. [37] includes 21,623 annotated nuclei boundaries. Due to this dataset being taken from multiple centers and including a diversity of nuclei appearances from multiple organs, segmentation models trained on it are likely to generalize well and can be better transferred to other HE-stained images. An example of the pathological image and nuclei annotation contained in the dataset is shown in Figure 4.

### 4.3. Breast Cancer Classification Results

**Figure 5:**
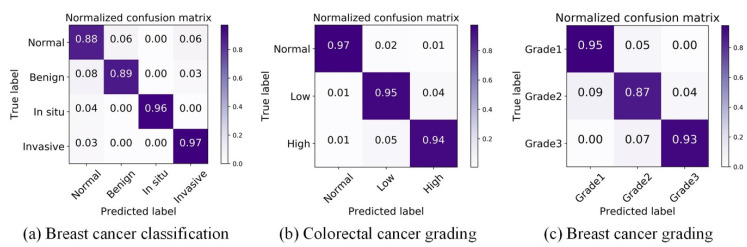
Visualization of normalized confusion matrix on three datasets.

**Figure 6:**
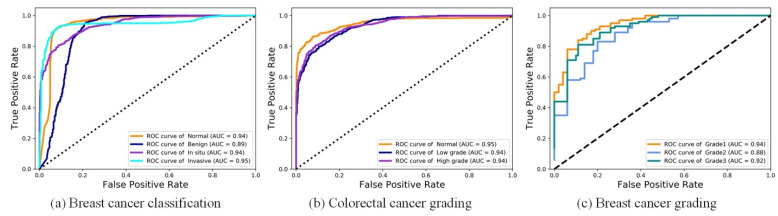
Visualization of ROC on three datasets.

We first conducted experiments on the breast cancer classification task to verify the effectiveness of DANet. For the four-class BC classification, based on the dataset published by Yan et al. [42], the proposed framework achieved an average accuracy of 92.5%. The proposed framework refers to: the patch-wise method (the DANet with Xception backbone) + the image-wise method (majority voting). Due to the high resolution of images contained in the original dataset, the current best practice is to divide the image into patches of the same size. The patch classification results are obtained by the patch-wise method first, and then the classification results of all the patches are fused in order to obtain the image-wise classification results. To focus on evaluating the patch-wise classification performance of the DANet, the image-wise method was fixed as the simplest majority voting (MV). Even with such a simple image-wise method, the proposed framework still achieved a higher classification accuracy (see Table 1) than the state-of-the-art method proposed by Yan et al. [42]. In addition, we also made comparisons with the classic CNN: ResNet50 and Xception. Similar to the above, the MV method was also used in the image-wise stage. The overall patch-wise classification performance of the DANet was demonstrated by the confusion matrix and ROC curve, as shown in Figure 5a and Figure 6a.

### 4.4. Colorectal Cancer Grading Results

Cancer grading is a fine-grained classification task, which faces more difficulties and challenges than cancer classification. However, the DANet is still effective at cancer grading. We conducted experiments on the tasks of breast cancer grading and colorectal cancer grading to verify the effectiveness of the DANet. In this subsection, we first introduce the experimental results of colorectal cancer grading, and the experimental results of breast cancer grading are introduced in the next subsection.

For the three-class CRC grading, the state-of-the-art method was proposed by Shaban et al. [49], which can capture image context information. It achieves an average accuracy of 95.70%. Since the image resolution is too high (4608 × 7680), utilizing the contextual information of an image is crucial for the algorithm. However, only using the simplest majority voting (MV) as the image-wise method, the proposed method still achieved almost the same accuracy (95.33%) as Shaban et al. [49]. This further illustrates the superiority of the DANet. In addition, we also made comparisons with the classic CNN: ResNet50 and Xception. The results of comparative experiments with other methods are shown in Table 2. The overall patch-wise classification performance of the DANet is demonstrated by the confusion matrix and ROC curve, as shown in Figure 5b and Figure 6b.

### 4.5. Breast Cancer Grading Results

For the three-class BC grading, the state-of-the-art method Nuclei-Aware Network (NANet) was proposed by Yan et al. [11], which can learn more nuclei-related features. It achieves an average accuracy of 92.20%. The DANet achieved almost the same classification effect as the NANet (see Table 3). We speculate that this is mainly due to the branch fusion attention of the DANet; the DCCA loss we used can maximize the correlation between the main branch (input is the original image) and the auxiliary branch (input is the nuclear image), and extract the common component of the two branches. This process plays the same role as the NANet in emphasizing the “nuclei-related features”. However, the limitation of the NANet is that it can only be effective at tasks that need to emphasize “nuclei-related features” (such as breast cancer grading) and cannot be applied to other pathological image classification tasks. However, the DANet is applicable to all tasks. This is mainly due to the branch selection attention mechanism, which performs adaptive selection of the feature representation learning on all decomposed branches according to different tasks. The overall patch-wise classification performance of the DANet is demonstrated by the confusion matrix and ROC curve, as shown in Figure 5c and Figure 6c.

### 4.6. Nuclei Segmentation Results

**Figure 7:**
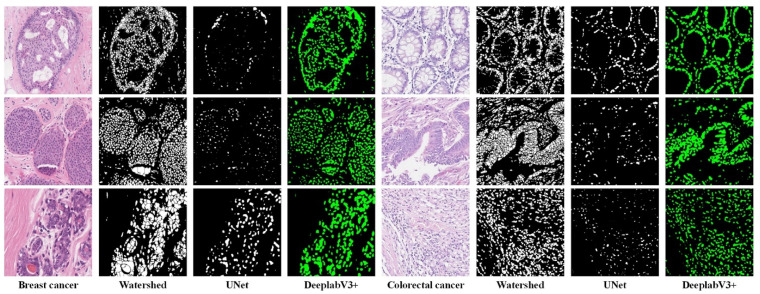
Nuclei segmentation results using different methods on two datasets. The Watershed method leads to merged nuclei (over-segmentation) and the UNet method leads to fragmented nuclei (under-segmentation).

To select the suitable method for nuclei segmentation, we compared three methods: Watershed, UNet [51], and DeepLabV3+ [39]. Watershed (Fiji version [52]) is the representative traditional image segmentation method, and UNet and DeeplabV3+ are the representative deep-learning-based image segmentation methods. The segmentation results (see Figure 7) of Watershed are too rough, and UNet has a serious under-segmentation problem. DeeplabV3+ is suitable for the nuclei segmentation task and obtains satisfactory segmentation results.

We only performed a visual qualitative analysis of the segmentation results because we did not have nuclear boundary annotations for the pathological image classification dataset. In addition, traditional indicators such as mIOU could not measure the segmentation results we needed. For example, we think that a slightly larger segmentation that includes the edge background of the nuclei may be better. The dataset proposed by Kumar et al. [37] was used to train a segmentation network, and the trained segmentation network was used to perform nuclei segmentation on our dataset.

### 4.7. Ablation Study

We further conducted ablation studies on different components of the proposed DANet. The experiments discussed in this section were focused on the breast cancer (BC) grading dataset. Each experiment was performed five times and a mean ± standard deviation is reported. We used the following metrics to evaluate the performance: accuracy, sensitivity, specificity, F-score, and the area under the receiver operating characteristic curve (AUC).

First of all, we fixed the other components of the proposed method, and verified the effectiveness of the fusion block and DCCA loss. The experimental results are shown in Table 4. After using the nuclei segmentation method to decompose a pathological image into two parts: the nuclei image and non-nuclei image, we first used Xception to verify the classification accuracy using only a single decomposed image. Compared with using the original pathological image for BC grading, the classification accuracy of using only a non-nuclei image was greatly reduced, and the classification accuracy of using only a nuclei image was slightly reduced. This illustrates that the pathological image is complex, and the nuclei or non-nuclei images alone are not sufficient to represent pathological image.

Compared with using only the original pathological image for BC grading, the classification accuracy of the DANet without the fusion block and DCCA loss was only slightly improved (+1.3%). After using the fusion block, the performance of the network was greatly improved (+7.5%). This shows that with the help of the fusion block, the DANet learned the feature representation that could not be discovered just by single branch learning.

One step further, after using DCCA loss on the basis of the fusion block, the overall performance of the network was further improved (+2.3%). For the task of BC grading, pathologists classify according to the three indicators of the Nottingham Grading System, which are all about nuclei-related features. In the DANet, the DCCA constraints act as branch fusion attention in the feature fusion of different branches. The middle branch obtained after the fusion of the main and down branches (containing only the nuclei) under DCCA constraints can focus on to the “nuclei-related features”. We speculate that this is the main reason for the improved classification performance after using the DCCA constraint.

Secondly, we fixed the other components of the proposed method (DANet without branch selection attention module), and verified the effectiveness of the branch selection attention module. The experimental results are shown in Table 5. Through the previous decomposition and fusion module, five branches obtained five feature vectors. We need to integrate these five feature vectors to obtain an overall feature representation of a pathological image. Commonly used integration methods include vector concatenate, max pooling, and mean pooling. The branch selection attention module achieved better results than these methods. We believe the importance of decomposed branches for different classification tasks varies. The branch selection attention module gives different attention weights to different branches through end-to-end learning. In contrast, the concatenate method is equivalent to always giving the same weight to the five branches, and the weights of the max and mean methods are not trainable, so the effect is not as good as the attention mechanism, which adaptively learns weights.

Finally, we compared the impact of different CNN backbones on the DANet. The experimental results are shown in Table 6. We first compared Xception with the most commonly used ResNet50 and Inception-V3, DANet with Xception as CNN backnone achieved better performance. Although classification performance was the focus of our study, we also considered the model size and computational cost. Therefore, we also compared Xception with the lightweight network architecture MobileNet-V2 [53], and there was only a small difference in their classification performance. This may be attributed to both Xception and MobileNet being based on depth-wise convolution. In actual use, MobileNet-V2 is also a good choice for CNN Backbone.

## 5. Conclusions

In this paper, we proposed a Divide-and-Attention network for HE-stained pathological image classification. We integrated image semantic segmentation into the framework of image classification. The results of the segmentation were used to decompose the target region of the original image. With such decomposed images, the DANet first performs feature learning independently in each branch, and then focuses on the most important feature representation through branch selection attention. In this way, the DANet is able to learn more representative features with respect to different structures and adaptively focus on the most important ones. In addition, for better feature learning, we introduced deep canonical correlation analysis (DCCA) loss to maximize the correlation of different branches and extract common components, which act as the branch fusion attention. Extensive experiments on different pathological image datasets showed that the proposed framework achieved competitive results. The DANet achieved an average classification accuracy of 92.5% in breast cancer classification, 95.33% in colorectal cancer grading, and 91.6% in breast cancer grading tasks, respectively. The paradigm behind Divide-and-Attention is general, which can be extended to other image analysis problems.

## Figures and Tables

**Table 1:** Comparison with the previous methods on the BC classification dataset.

Methods (BC-Classification)	Accuracy (%)	AUC
Vang et al. [46]	87.5	-
Golatkar et al. [47]	85.0	-
Yan et al. [42]	91.3	0.89
ResNet50 [48] + MV	84.9	0.85
Xception [40] + MV	85.7	0.86
Ours (DANet + MV)	92.5	0.93

**Table 2:** Comparison with the previous methods on the CRC grading dataset.

Methods (CRC-Grading)	Accuracy (%)	AUC
Awan et al. [44]	90.66	-
Hou et al. [50]	92.12	-
Shaban et al. [49]	95.70	-
ResNet50 [48] + MV	92.08	0.90
Xception [40] + MV	92.09	0.91
Ours (DANet + MV)	95.33	0.94

**Table 3:** Comparison with the previous methods on the BC grading dataset.

Methods (BC-Grading)	Accuracy (%)	AUC
Wan et al. [10]	69.0	-
Yan et al. [11]	92.2	0.92
ResNet50 [48]	81.3	0.83
Xception [40]	81.8	0.85
Ours (DANet)	91.6	0.91

**Table 4:** Ablation study on the fusion block (FB) and DCCA loss.

Items	Accuracy (%)	Sensitivity (%)	Specificity (%)	F-Score (%)	AUC
Pathology only (Xception)	81.8 ± 0.2	81.1 ± 0.2	82.7 ± 0.3	81.2 ± 0.3	0.85 ± 0.08
Nuclei only (Xception)	79.2 ± 0.3	79.4 ± 0.2	79.1 ± 0.3	79.2 ± 0.3	0.83 ± 0.07
Non-nuclei only (Xception)	70.1 ± 0.4	68.3 ± 0.3	70.5 ± 0.4	69.6 ± 0.4	0.72 ± 0.12
DANet w/o FB and DCCA	83.1 ± 0.2	82.5 ± 0.3	85.2 ± 0.2	82.0 ± 0.5	0.86 ± 0.06
DANet w/o DCCA	89.3 ± 0.1	88.3 ± 0.2	89.8 ± 0.1	88.8 ± 0.3	0.90 ± 0.03
DANet	91.6 ± 0.3	91.5 ± 0.2	92.1 ± 0.1	91.4 ± 0.3	0.91 ± 0.02

**Table 5:** Ablation study on branch selection attention module.

Items	Accuracy (%)	Sensitivity (%)	Specificity (%)	F-Score (%)	AUC
Mean	80.8 ± 0.9	81.5 ± 0.5	81.1 ± 0.5	81.0 ± 0.4	0.81 ± 0.06
Max	83.6 ± 0.6	82.2 ± 0.4	84.0 ± 0.6	82.5 ± 0.4	0.82 ± 0.08
Concat	84.5 ± 0.3	82.9 ± 0.4	85.3 ± 0.3	83.1 ± 0.5	0.84 ± 0.06
Attention	91.6 ± 0.3	91.5 ± 0.2	92.1 ± 0.1	91.4 ± 0.3	0.91 ± 0.02

**Table 6:** Ablation study on CNN backbones.

Items	Accuracy (%)	Sensitivity (%)	Specificity (%)	F-Score (%)	AUC
ResNet50	89.5 ± 0.6	90.9 ± 0.5	91.1 ± 0.6	89.9 ± 0.5	0.90 ± 0.05
Inception-V3	89.8 ± 0.4	90.5 ± 0.6	88.5 ± 0.3	89.7 ± 0.5	0.89 ± 0.02
MobileNet-V2	91.2 ± 0.2	90.4 ± 0.2	92.3 ± 0.1	91.0 ± 0.3	0.92 ± 0.03
Xception	91.6 ± 0.3	91.5 ± 0.2	92.1 ± 0.1	91.4 ± 0.3	0.91 ± 0.02

## Data Availability

Not applicable.
